# The prevalence and predictor factors of urinary tract infection in type 2 diabetes mellitus patients receiving SGLT2 inhibitors in Qatar: A retrospective cohort study

**DOI:** 10.1080/20523211.2026.2645913

**Published:** 2026-04-24

**Authors:** Shahad H. Alhefel, Binny Thomas, Mohamed Sherbash, Sara Hayder, Mohamed Izham Ibrahim, Rajvir Singh, Ahmad H. Alhefel, Salam Abou Safrah, Ahmed Karawia, Ameena Alyazeedi

**Affiliations:** a Department of Pharmacy, Hamad Medical Corporation, Doha, Qatar; b College of Pharmacy, QU Health, Qatar University, Doha, Qatar; c General Physician, Ministry of Public Health, Doha, Qatar

**Keywords:** SGLT2 inhibitors, Urinary tract infection (UTI), Type 2 diabetes mellitus (T2DM), Older adults

## Abstract

**Background and aims:** Urinary Tract Infections (UTIs) are common in Type 2 Diabetes Mellitus (T2DM) patients. While Sodium-Glucose Co-Transporter 2 (SGLT2) inhibitors improve T2DM treatment by lowering blood glucose, they may increase UTI risk. Research findings on this association are mixed. This study aims to determine UTI prevalence and identify risk factors in elderly T2DM patients using SGLT2 inhibitors, to enhance treatment strategies and patient safety.

**Methods:**

A retrospective cohort study was conducted at two hospitals to evaluate UTI prevalence and predictors in elderly T2DM patients treated with SGLT2 inhibitors. Data from electronic health records over one year were analysed. Patients aged 60 + with a UTI diagnosis within 90 days of starting SGLT2 inhibitors were included. The primary outcome was UTI prevalence; secondary outcomes included factors like age, gender, comorbidities, HbA1c levels, and kidney function.

**Results:**

In a cohort of 1,024 elderly T2DM patients, 49% experienced UTIs. Significant predictors included gender, nationality, and age. Females had a higher UTI prevalence (68% vs. 32%, *p* < 0.001), and Qatari patients had a higher UTI prevalence compared with non-Qatari patients (63% vs. 37%, *p* = 0.03). Older age was associated with higher UTI risk (*p* < 0.001). There were no significant associations with HbA1c levels, creatinine levels, or medication duration. Logistic regression confirmed females and older age as significant UTI predictors. These findings highlight demographic factors’ importance in UTI risk among T2DM patients on SGLT2 inhibitors.

**Conclusion:**

The study identified varying risks of urinary tract infections (UTIs) among users of SGLT2 inhibitors, influenced by factors like gender, age, and existing health conditions. This underscores the need for cautious prescribing of SGLT2 inhibitors, emphasising personalised risk assessment and careful evaluation of benefits versus risks. Future research should focus on refining UTI risk prediction and developing targeted interventions for this patient population.

## Background

Urinary Tract Infections (UTIs) are a prevalent complication in individuals with Type 2 Diabetes Mellitus (T2DM) (American Diabetes Association, 2020). The introduction of Sodium-Glucose Co-Transporter 2 (SGLT2) inhibitors has significantly transformed T2DM treatment strategies by promoting glycosuria and reducing blood glucose levels, these agents have demonstrated substantial benefits in the management of heart failure – reflected by their inclusion in treatment guidelines across many European countries – and emerging evidence suggests they may also offer neuroprotective effects (Berhan & Barker, 2013; Birkeland et al., 2017; Borovac et al., 2022). Nevertheless, the use of SGLT2 inhibitors has been associated with an increased incidence of UTIs, prompting extensive research to investigate the prevalence and predictors of this comorbidity. Moreover, there is a well-established link between SGLT2 inhibitor therapy and genital tract infections, particularly mycotic infections such as vulvovaginal candidiasis in women and balanitis in men (Johnsson et al., 2013). This is primarily attributed to the mechanism of action of these agents, which promote glucosuria and thereby create an environment conducive to microbial growth in the genitourinary tract. Multiple studies have reported a higher risk of genital tract infections with SGLT2 inhibitor use with meta-analyses showing incidence rates ranging from 4% to 6%, especially among females and uncircumcised males (Caro et al., 2022).

Various studies have delved into the relationship between SGLT2 inhibitors and UTIs in T2DM patients, presenting mixed and sometimes contradictory results. Some research highlights an increased risk of UTIs linked to glucosuria and glycosuria induced by SGLT2 inhibitors, arguing that glucose in the urine fosters an environment conducive to bacterial growth (Caro et al., 2022; Confederat et al., 2023), thereby elevating UTI susceptibility. On the contrary, other analyses suggest that the incidence of UTIs among patients treated with SGLT2 inhibitors does not significantly differ from those receiving other antidiabetic treatments (Confederat et al., 2023). These reports propose that the increased urine output caused by SGLT2 inhibitors might aid in flushing out bacteria, potentially lowering the risk of UTIs (Dave et al., 2019).

To reconcile these conflicting findings, it is crucial to consider a range of factors that could influence UTI prevalence in T2DM patients treated with SGLT2 inhibitors, such as age, sex, duration of diabetes, glycemic control, renal impairment, and previous UTI history. Additionally, variations in study methodologies, including design, sample size, participant demographics, and differing UTI definitions, must be acknowledged (Borovac et al., 2022; Dave et al., 2019).

Despite growing global evidence, data from the Middle East remain limited, and regional patient characteristics – including high diabetes prevalence, distinct comorbidity patterns, climatic influences, and healthcare utilisation behaviours – may alter observed outcomes. Addressing this gap represents a key novelty of the present study. By examining real-world patients from a local population, this investigation provides region-specific evidence that complements existing international literature and enhances external validity (Dave et al., 2019; DeFronzo et al., 2017; Fadini et al., 2017).

Furthermore, beyond estimating incidence, there is increasing clinical value in identifying patients at elevated risk. Predictive modelling offers a temporal benefit by enabling early risk stratification, individualised monitoring, and proactive preventive strategies. Incorporating a predicting algorithm into the analytical framework allows translation of observational findings into practical clinical decision-support tools (Kaku et al., 2014).

This research holds importance for several reasons. Firstly, it aims to clarify the ambiguity surrounding the prevalence of UTIs among T2DM patients on SGLT2 inhibitor therapy, thereby contributing valuable insights that could fill existing gaps in the literature and provide region-specific information. Secondly, given the general prominence of UTIs, especially in older populations, and the reported benefits and potential UTI risks associated with SGLT2 inhibitors, this study seeks to elucidate the extent of this issue and its implications for patient outcomes. Thirdly, identifying predictors of UTIs in patients receiving SGLT2 inhibitors could enable healthcare providers to implement preventive measures and closely monitor those at high risk, enhancing patient safety. Lastly, the findings of this research could support healthcare professionals in making informed treatment decisions regarding the use of SGLT2 inhibitors for T2DM patients, allowing for customised treatment plans that minimise infection risks.

The objective of this study is to examine the prevalence of UTIs and identify predictive factors among elderly T2DM patients undergoing treatment with SGLT2 inhibitors. By addressing discrepancies in existing literature and identifying potential risk factors associated with UTIs, this research endeavours to improve the understanding of the implications of SGLT2 inhibitor therapy in the management of T2DM, representing a novel investigation within the Qatari setting.

## Methods

### Study design

A retrospective cohort study was conducted to evaluate the prevalence and predictive factors of urinary tract infections (UTIs) in patients with Type 2 Diabetes Mellitus (T2DM) receiving Sodium-Glucose Cotransporter-2 (SGLT2) inhibitors as part of their diabetes management.

### Study setting

The study was carried out at Rumailah Hospital and Hamad General Hospital, focusing on the geriatric population. Data were obtained from electronic health records (EHRs) maintained by these hospitals.

### Study population

The study population included T2DM patients aged 60 years and older who were treated with SGLT2 inhibitors (dapagliflozin, and empagliflozin) available at Hamad Medical Corporation (HMC) during the study period. Inclusion criteria consisted of patients who had a UTI diagnosis within 90 days of initiating SGLT2 inhibitor therapy. Patients were excluded if they were on dialysis, had other forms of diabetes, had incomplete clinical laboratory data, or had received a kidney transplant.

### Data collection

Data were retrospectively collected over one year, from January 1, 2023, to December 31, 2023. Information was extracted using Cerner from both inpatient and outpatient clinic records. All patients meeting the inclusion criteria within the specified timeframe were included in the study. Based on an estimated UTI prevalence of 50%, a sample size of approximately 400 patients was deemed sufficient, with a 5% margin of error and a 95% confidence interval.

### Variables and measures

Potential confounding variables and predictors included:

Demographic Data: Gender, race/ethnicity, age.

Clinical Data: Cardiovascular events, overweight status, baseline glomerular filtration rate (GFR), HbA1c average, and use of other anti-diabetic medications.

These variables were obtained from the Medical Record Number (MRN) and patient folders and entered into a Microsoft Excel database for subsequent analysis.

### Outcome measures

#### Primary outcome

The primary outcome measure was the prevalence of UTIs among T2DM patients treated with SGLT2 inhibitors, defined as the proportion of patients experiencing at least one UTI during the study period.

#### Secondary outcome

The secondary outcome involved identifying factors predictive of UTI occurrence, which included:

Age: Determining susceptibility across different age groups.

Gender: Examining gender-related differences in UTI prevalence.

Comorbidities: Evaluating the impact of comorbid conditions such as kidney disease or hypertension.

HbA1c Levels: Investigating the association between higher HbA1c levels and UTI incidence.

Kidney Function: Analysing the relationship between kidney function and UTI occurrence.

### Statistical analysis

Statistical analyses were performed using SPSS Version 29. Both descriptive and inferential statistics were utilised with a significance level set at 0.05 (two-tailed).

Descriptive Statistics: Means, medians, and standard deviations were calculated for continuous variables. Frequencies and percentages were computed for categorical variables. The overall UTI prevalence was calculated by dividing the number of UTI cases by the total number of patients in the cohort.

Bivariate Analysis: Chi-square test was applied for categorical variables to identify significant associations between predictor factors and UTI occurrences. For continuous variables, the T-test test was used to compare means between positive and negative UTI infection.

Multivariable Analysis: Logistic regression analysis was conducted to identify predictors of UTI. The dependent variable was UTI occurrence (binary: 0/1 for No/Yes), while independent variables included demographic and clinical factors. Odds ratios (OR) with 95% confidence intervals (CI) were calculated, with an OR > 1 indicating a higher likelihood of UTI occurrence.

Confounding Control: Covariate adjustment was employed in the regression analysis to assess the relationship between SGLT2 inhibitor use and UTI occurrence while controlling for potential confounders. Multiple imputation techniques were applied to address any missing data.

### Ethical approval

This study was carried out by the Helsinki Declaration principles and was approved by the HMC Medical Research Centre MRC-01-23-703. Informed consent was waived by IRB.

## Results

Baseline Demographics and Clinical Characteristics

In our comprehensive analysis of 1,024 participants, the distribution of demographic and clinical characteristics revealed insightful trends. The cohort consisted of 59% females (*n* = 602) and 41% males (*n* = 422), with a majority (60%, *n* = 612) being Qatari nationals, and the remaining 40% (*n* = 412) were non-Qatari. The non-Qatari group included individuals from diverse ethnic and geographic backgrounds, reflecting Qatar’s multinational population. The participants were predominantly older adults, with an average age of 73 years (SD = 6).

Regarding clinical characteristics, urinary tract infection (UTI) culture results showed an almost even distribution between negative (51%, *n* = 527) and positive outcomes (49%, *n* = 497). The prevalence of coronary artery disease (CAD) was observed in 31% (*n* = 319) of the participants, while a significant majority did not have CAD (69%, *n* = 705). Hypertension (HTN) was highly prevalent, with 90% (*n* = 919) of the participants being hypertensive, compared to 10% (*n* = 105) who were not. Heart failure (HF) was less common, affecting 17% (*n* = 171) of the participants, whereas 83% (*n* = 853) did not have HF. Chronic kidney disease (CKD) was present in 37% (*n* = 382) of the cohort, while 63% (*n* = 642) did not have CKD.

The survival status indicated that 95% (*n* = 975) of the participants were alive, whereas 5% (*n* = 49) were deceased. Clinical measures showed an average HbA1c level of 8% (SD = 1), a height of 160 cm (SD = 12), and a weight of 82 kg (SD = 20). The average creatinine level was reported at 106 μmol/L (SD = 68), and the Albumin/Creatinine ratio (A/C) was 5 (SD = 20). The urine creatinine level averaged 5,573 μmol/L (SD = 3,944), highlighting significant renal function variability. Additionally, participants had been on medication for an average of 21 months (SD = 19), indicating a varied duration of treatment across the sample. Statistical analysis was conducted using pooled t-tests for mean values and Pearson's chi-squared tests for frequency distributions, underscoring the methodological rigor of our study (Table 1).

**Table 1.** Distribution of Demographic and clinical characteristics.

N	1,024
Sex	
Female	602 (59%)
Male	422 (41%)
Nationality	
Non-Qatari	412 (40%)
Qatari	612 (60%)
Age	73 (6)
Result value	
Negative	527 (51%)
Positive	497 (49%)
Co-morbidities	
CAD	
No	705 (69%)
Yes	319 (31%)
HTN	
No	105 (10%)
Yes	919 (90%)
HF	
No	853 (83%)
Yes	171 (17%)
CKD	
No	642 (63%)
Yes	382 (37%)
Status	
Alive	975 (95%)
Dead	49 (5%)
HBA1C	8 (1)
Height	160 (12)
Weight	82 (20)
Creatinine	106 (68)
A/C:Albumin/Creatinine	5 (20)
U creatinine	5,573 (3,944)
Months After Medication (Months)	21 (19)

Abbreviations: SCr, serum creatinine; SGLT2, sodium-glucose cotransporter-2; eGFR, estimated glomerular filtration rate; HbA1c,  glycated hemoglobin; BMI, Body mass index; BSA, body surface area.

### Main analysis

#### Primary outcomes

Table 2 presents the association of urinary tract infection (UTI) outcomes with various independent variables among our cohort of 1,024 participants, highlighting significant associations and trends.

**Table 2.** Association of UTI & Other Independent Variables.

	Negative	Positive	Total	Test
N	527 (51%)	497 (49%)	1,024 (100%)	
Sex				
Female	265 (50%)	337 (68%)	602 (59%)	<0.00
Male	262 (50%)	160 (32%)	422 (41%)	
Nationality				
Non-Qatari	229 (43%)	183 (37%)	412 (40%)	0.03
Qatari	298 (57%)	314 (63%)	612 (60%)	
Age	72 (6)	73 (7)	73 (6)	<0.00
HBA1C	8 (1)	8 (1)	8 (1)	0.85
Creatinine	107 (65)	105 (71)	106 (68)	0.67
U creatinine	6,089 (4,049)	5,027 (3,756)	5,573 (3,944)	<0.00
Ualbulmin	35 (100)	43 (152)	39 (128)	0.34
A/C:Albumin/Creatinine	4 (14)	6 (24)	5 (20)	0.10
Months After Medication (Months)	20 (19)	21 (19)	21 (19)	0.58
Height	161 (11)	158 (12)	160 (12)	<0.00
Weight	83 (19)	81 (21)	82 (20)	0.22

UTI outcomes were almost evenly split, with 51% (*n* = 527) testing negative and 49% (*n* = 497) testing positive. A significant gender disparity was observed in UTI positivity: 68% (*n* = 337) of positive cases were female, compared to 32% (*n* = 160) in males, yielding a *p*-value of (<0.001). Nationality also influenced UTI outcomes, with Qatari nationals showing a slightly higher incidence of positive UTI results (63%, *n* = 314) compared to non-Qataris (37%, *n* = 183), with a *p*-value of (0.03). Age showed a subtle but statistically significant difference between UTI negative and positive groups, with an average age of 72 years (SD = 6) for negative outcomes and 73 years (SD = 7) for positive outcomes, supported by a *p*-value of (<0.001). Notably, no significant differences were found in HbA1c levels between UTI outcomes (*p* = 0.85), creatinine levels (*p* = 0.67), or weight (*p* = 0.22). Urine creatinine levels showed a significant difference between the negative and positive UTI groups, with higher levels observed in the negative group (6,089 vs. 5,027, *p* < 0.00),

The analysis of the Albumin/Creatinine ratio and the duration of medication use did not reveal significant associations with UTI outcomes, with *p*-values of (0.10 and 0.58), respectively.

#### Secondary outcomes

The multivariate logistic regression analysis elucidated the multifactorial nature of urinary tract infection (UTI) susceptibility within our cohort, quantifying the impact of demographic, clinical, and comorbidity variables on UTI risk, both before and after adjustment for potential confounders. Sex was confirmed as a significant determinant of UTI risk. Males exhibited markedly reduced odds of infection compared to females, as evidenced by both unadjusted (OR = 0.48, 95% CI: 0.37–0.61, *p* < 0.001) and adjusted odds ratios (OR = 0.49, 95% CI: 0.35–0.67, *p* < 0.001). Nationality initially appeared to influence UTI risk, with an unadjusted OR of 1.31 (*p* = 0.03). However, this association lost significance upon adjustment (OR = 1.28, *p* = 0.38).

Age emerged as a consistent predictor of increased UTI risk, with both unadjusted (OR = 1.04, 95% CI: 1.02–1.06, *p* < 0.001) and adjusted models (OR = 1.03, 95% CI: 1.01–1.05, *p* = 0.002) indicating that older age was associated with higher UTI risk. Urine creatinine levels showed a marginal but statistically significant association with UTI risk in the adjusted model (OR = 1.00, 95% CI: 1.00–1.01, *p* = 0.01). The presence of coronary artery disease (CAD) and heart failure (HF) was initially posited as exacerbating factors for UTI risk. However, only CAD retained borderline significance in the adjusted analysis (OR = 1.33, 95% CI: 1.00–1.76, *p* = 0.05), while the association with HF was not statistically significant post-adjustment (OR = 1.35, 95% CI: 0.93–1.95, *p* = 0.11) (Table 3).

**Table 3.** Multivariate Logistic Regression of Predictors for UTI in T2DM Patients on SGLT2 Inhibitors.

Unadjusted				Adjusted		
Variable	Odds ratio	*P*-value	95%Cl	Odds ratio	*P*-value	95%Cl
Sex						
Male	0.48	0.001	0.37–0.61	0.49	0.001	0.35–0.67
Nationality						
Qatari	1.31	0.03	1.02–1.69	1.28	0.38	0.85–1.48
Age	1.04	0.001	1.02–1.06	1.03	0.002	1.01–1.05
HBA1C	0.99	0.84	0.91–1.07	0.98	0.60	0.89–1.06
Creatinine	0.99	0.67	0.99–1.00	1.00	0.61	0.98–1.01
U creatinine	0.99	0.001	0.99–1.00	1.00	0.01	0.99–1.00
Ualbulmin	1.00	0.35	0.99–1.00	1.00	0.58	0.99–1.00
A/C: Albumin/Creatinine	1.00	0.11	0.99–1.01	1.00	0.69	0.99–1.01
Co-morbidity						
CAD	1.31	0.04	1.01–1.71	1.33	0.05	.099–1.79
CKD	0.96	0.75	0.74–1.23	0.88	0.46	0.64–1.22
HTN	1.40	0.10	0.93–2.12	1.39	0.13	0.90–2.14
HF	1.66	0.003	1.19–2.23	1.35	0.11	0.92–1.97
Months After Medication (Months)	1.00	0.57	0.99–1.00	1.00	0.38	0.99–1.01
Height	0.97	0.001	0.96–0.99	1.00	0.87	0.98–1.01
Weight	0.99	0.21	0.98–1.00	1.00	0.65	0.99–1.00

## Discussion

The prevalence of urinary tract infections (UTIs) in patients receiving sodium-glucose co-transporter-2 inhibitors (SGLT2i) in our study revealed an almost even distribution between negative (51%, *n* = 527) and positive (49%, *n* = 497) UTI culture results. Previous studies have reported variable UTI rates in patients on SGLT2i, ranging from increased risk to negligible differences compared to non-users (Lin et al., 2022). This near-equal split indicates that while SGLT2i therapy is linked to a considerable number of UTI cases, the risk may not be as heightened or universally experienced across all patient populations as previously thought (Lin et al., 2022). These findings highlight the necessity for vigilant patient monitoring and the development of tailored preventive strategies to effectively balance the therapeutic benefits of SGLT2i with their potential adverse effects, particularly in individuals at higher risk for UTIs.

Our analysis reveals significant associations between UTI risk and variables such as sex, nationality, age, and urine creatinine levels within our cohort. These findings underscore the complexity of UTI risk factors and highlight the need for targeted research and intervention strategies that consider these demographic and clinical nuances (Tanaka et al., 2015).

A significant gender disparity in UTI positivity was observed, with females exhibiting a higher risk of UTIs compared to males. This finding aligns with existing literature, which attributes increased UTI risks in females to anatomical and hormonal factors (Kang et al., 2021). The consistently higher prevalence of UTIs among females in our cohort emphasises the importance of developing gender-specific prevention and management strategies for UTIs (Caro et al., 2022). Nationality also played a role in UTI outcomes, with Qatari nationals showing a slightly higher incidence of positive UTI results compared to non-Qataris. This difference suggests a statistically significant association between nationality and UTI positivity in our study population. The non-Qatari group included individuals from diverse ethnic and geographic backgrounds, reflecting Qatar’s multinational population. This classification was based solely on nationality and does not indicate biological uniformity. Potential differences in genetic background, lifestyle factors, disease prevalence, and body composition may exist; however, the study was not designed to evaluate ethnicity-specific variations. Further investigation into socio-cultural, genetic, or environmental factors that may influence these outcomes is warranted to better understand and address the disparities observed. Age showed a subtle but statistically significant difference between UTI-negative and UTI-positive groups, with older age slightly associated with increased UTI risk (Confederat et al., 2023; Lardaro et al., 2024). Although the clinical relevance of this one-year difference is limited, it suggests that age may be a contributing factor to UTI risk, meriting further exploration into how aging processes impact susceptibility to infections (Lardaro et al., 2024).

Notably, no significant difference was found in HbA1c levels between UTI outcomes, indicating that glycemic control, as measured by HbA1c, does not have a statistically significant association with UTI risk in this cohort. some studies suggest poor glycemic control increases UTI risk, while others find no significant correlation (Mascolo et al., 2022; Rawshani et al., 2018). Our results contribute to the latter, indicating other factors might be more critical in UTI development among T2DM patients on SGLT2i. However, urine creatinine levels showed a significant difference between the negative and positive UTI groups, with higher levels observed in the negative group. This suggests that urine creatinine might be inversely related to UTI risk, potentially indicating better renal function or other protective mechanisms in patients with higher urine creatinine levels. Physical attributes such as height and weight did not significantly influence UTI risk after adjustment, suggesting other clinical and demographic factors play a more critical role. Further studies are needed to elucidate the potential underlying factors linking height to UTI susceptibility. The analysis of the Albumin/Creatinine ratio and the duration of medication use did not reveal significant associations with UTI outcomes, suggesting that these factors do not markedly influence UTI risk in our population. This indicates that while certain demographic and clinical variables are significant predictors of UTI risk, others may have a negligible impact, emphasising the need for a multifaceted approach in UTI risk assessment and management (Zhou et al., 2019).

Multivariate logistic regression analysis delineates a landscape where gender, age, and, to a lesser extent, certain comorbidities significantly modulate UTI risk. These insights advocate for a stratified approach to UTI prevention, underpinned by a nuanced understanding of the interrelations between demographic characteristics, clinical parameters, and comorbidity profiles. The analysis confirmed sex as a significant determinant of UTI risk, with males exhibiting markedly reduced odds of infection compared to females. This finding is consistent with established epidemiological trends and reinforces the imperative for gender-specific considerations in UTI risk mitigation strategies. Females’ higher risk of UTIs, likely due to anatomical and hormonal factors, necessitates targeted preventive measures in this demographic. Although nationality initially appeared to influence UTI risk, the significance of this association dissipated upon adjustment. This suggests that the initial observation may be attributed to confounding factors rather than a direct causal relationship. Thus, nationality may not be a primary risk factor when other variables are controlled for (Confederat et al., 2023). Age emerged as a consistent predictor of increased UTI risk across both unadjusted and adjusted models, indicating a linear relationship between advancing age and UTI susceptibility (Tahara et al., 2013; Tanaka et al., 2015). This age-related trend underscores the need for heightened vigilance and preventative measures among older populations, highlighting the importance of age-specific strategies in UTI prevention. Conversely, urine creatinine levels bore a marginal but statistically significant association with UTI risk in the adjusted model, hinting at the potential role of urinary biomarkers in refining risk stratification models for UTI. While the effect size was small, this finding suggests that higher urine creatinine levels might be inversely related to UTI risk, potentially reflecting better renal function or other protective mechanisms. The presence of coronary artery disease (CAD) and heart failure (HF) was initially posited as exacerbating factors for UTI risk; however, only CAD retained borderline significance in the adjusted analysis. The association with HF was not statistically significant post-adjustment. This nuanced observation suggests that while cardiovascular comorbidities may influence UTI risk, their role is likely modulated by a complex interplay of systemic factors (Mascolo et al., 2022; Rawshani et al., 2018).

Interestingly, the analysis did not substantiate a significant role for physical attributes such as height and weight in influencing UTI risk post-adjustment. These challenges preconceived notions about the potential protective or risk-modifying effects of body morphology on UTI susceptibility (Scheen, 2014). The lack of significant association with height and weight indicates that other factors may play a more critical role in determining UTI risk in our cohort.

## Strengths and Limitations

This study represents the first investigation in Qatar to assess the prevalence of urinary tract infections (UTIs) in elderly patients and those receiving treatment with SGLT2 inhibitors, demonstrating sufficient statistical power to support its findings. However, several limitations should be noted. The study was conducted retrospectively, which may introduce selection bias and affect patient inclusion and UTI detection rates. While the sample size was sufficient for primary analyses, the cohort may not fully represent all T2DM patients on SGLT2i in other populations or settings. Nevertheless, the inclusion of patients from diverse nationalities enhances the external validity of the findings and supports broader applicability within similar multiethnic populations. Additionally, the study duration was limited to one year, which may not fully capture long-term trends and outcomes.

## Conclusion

Our study identified a balanced distribution of urinary tract infections (UTIs) among patients using SGLT2 inhibitors, with factors such as gender, age, and comorbidities significantly influencing UTI incidence. This indicates that the risk of UTI is not uniform across all patients. Therefore, SGLT2 inhibitors are recommended to be used with caution, considering these predictive factors and carefully measuring the benefit-risk ratio for each patient. Further research is necessary to enhance UTI risk assessment and intervention methods for this population. This may include considering periodic urine cultures – even in asymptomatic patients – as a proactive approach to detect and manage subclinical infections in diabetic individuals receiving SGLT2 inhibitor therapy.
